# Thermodynamic irreversibility effects with Marangoni convection for third grade nanofluid flow

**DOI:** 10.1016/j.heliyon.2023.e16192

**Published:** 2023-05-12

**Authors:** Khursheed Muhammad, T. Hayat, Inayat Ullah, S. Momani

**Affiliations:** a Department of Humanities and Sciences, School of Electrical Engineering and Computer Science (SEECS), National University of Sciences and Technology (NUST), Islamabad, Pakistan; b Department of Mathematics, Quaid-I-Azam University 45320, Islamabad 44000, Pakistan; c Nonlinear Dynamics Research Center (NDRC), Ajman University, Ajman, United Arab Emirates

**Keywords:** Third grade fluid, Soret-Dufour effects, Marangoni convection, Thermal radiation, Activation energy, Entropy generation

## Abstract

In this study, an analysis was performed to investigate the thermal and mass transport of radiative flow of a third-grade nanofluid with magnetohydrodynamic. The analysis concerns two-dimensional flow around an infinite disk. Heat transport is studied via heat generation/absorption, thermal radiation and Joule heating. Chemical reaction with activation energy is also considered. The nanofluid characteristics, including Brownian motion and thermophoretic diffusion, are explored via the Buongiorno model. Entropy analysis is also conducted. Moreover, the surface tension is assumed to be a linear function of concentration and temperature. Through adequate dimensionless variables, governed PDEs are non-dimensionlized and then tackled by ND-solve (a numerical method in Mathematica) for solutions purposes. Entropy generation, concentration, velocity, Bejan number and temperature are plotted as functions of the involved physical parameters. It is noticed that higher Marangoni number intensify velocity however it causes a decrease in the temperature. Entropy rate and Bejan number boost for large value of diffusion parameter.

## 1 Introduction

In the current scenario, nanofluids become operative coolant media in many industrial and technological processes. Due to high thermal efficiency, nanofluid got incredible popularity among researchers and scientists. The idea of adding nanoparticles in traditional fluid for advancing its thermal conduction was given by Choi [1]. He was the first to propose that normal fluids can be replaced by more thermally effective fluids called nanofluids. Normal fluids have limited applications due to their poor thermal conductivity. The introduction of nanofluids brings a lot of new applications in industrial and mechanical research. Buongiorno [2] presented the mathematical model for nanofluid flow based on thermophoresis and Brownian diffusion. Alsaedi et al. [3] conducted a study on a hybrid nanofluid that is enclosed between two coaxial cylinders. Entropy generation and melting phenomena during flow of nanofluid is examined by Alsaadi et al. [4]. Kandasamy et al. [5] examined the MHD flow of nanomaterial via the Buongiorno model. Sheikholeslami and Shehzad [6] studied nanofluid flow saturated through a porous medium with mixed convection. Muhammad et al. [7] provided a study on a fourth-grade nanofluid subjected to both stagnation point and convective boundary conditions. The latest work in this area can be seen in Refs. [[8], [9], [10], [11], [12], [13]].

The Marangoni effect is due to the surface tension gradient, and it is the mass transfer between two fluid interfaces. Temperature-dependent aforementioned phenomena are known by thermo-capillary convection. James Thomson introduced this mechanism in 1855. It is widely used in the field of artwork. Soap film stabilization, convection cells or Benard cells, etc. are common applications of the Marangoni effect. Sreenivasulu et al. [14] expressed radiation impact in the MHD flow of viscous material with thermosolutal Marangoni convection. Hayat et al. [15] studied Marangoni convection and thermal radiation in flow of nanomaterial. Zhao et al. [16] examined the Soret and Dufour effects of fractional magneto hydrodynamic Maxwell fluid. Mahanthesh and Gireesha [17] consider Marangoni convection, joule heating, thermal radiation and viscous dissipation during the flow of Casson nanomaterial. Zhuang and Zhu [18] examined Marangoni convection in power-law nanoliquids. The energy flux generation via concentration gradient is initially presented by Dufour and called the thermal diffusion (Dufour) effect. Similarly, generation of mass flux by temperature gradient is referred as thermo-diffusion or Soret effect. Soret and Dufour impacts in MHD viscous fluid flow due to rotating cone with radiation effect is explored by Khan et al*.* [19]. Studies regarding the heat and mass transport are addressed in Refs. [[20], [21], [22], [23], [24]].

To efficient devices, scientists and researchers are constantly looking for procedures to control energy consumption. The main objective is to minimize heat loss to maximize the efficiency of machines. Entropy optimization plays a vital role in the improvement of the performance of many devices in various engineering and industrial sectors. Bejan [25] initially introduce the idea of entropy optimization. Govindaraju et al. [26] investigated the entropy optimization of MHD nanomaterial flow past a stretchable surface. Hayat et al. [27] investigated the entropy generation of Ree-Eyring nanomaterial flow bounded by rotating disks with activation energy. A few observations regarding entropy optimization are given in Refs. [23,[28], [29], [30], [31], [32]].

In the present work, our main intention is to examine the transportation of Marangoni convection in third-grade nanofluid with Dufour and Soret effects. Joule heating, thermal radiation, and internal heat generation describe heat transport features, while the chemical reaction is considered with activation energy. Governing equations (PDEs) are transformed by using appropriate variables. Entropy rate and Bejan number are considered. Effects of different flow parameters on quantities of interest are explored graphically.

## 2 Mathematical formulation

**Fig. (1):**
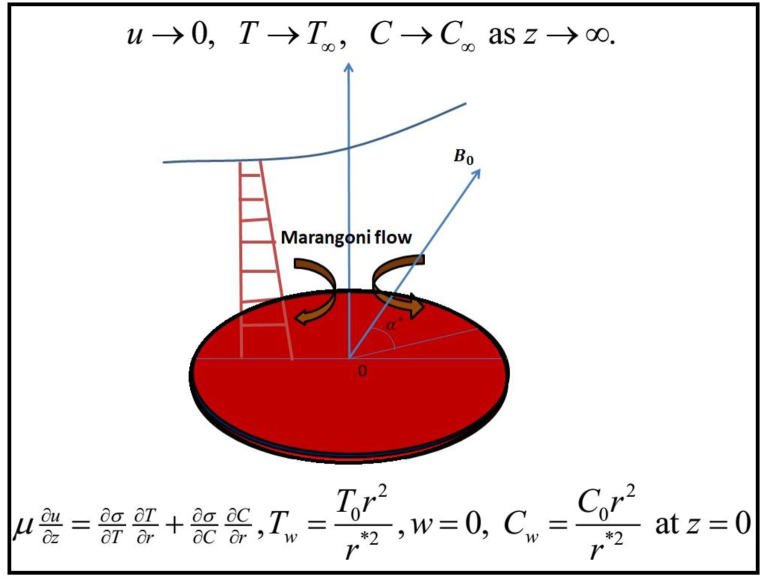
Flow geometry.

Marangoni convective inclined flow of third grade nanofluid is considered by an infinite disk. Buongiorno model for nanofluid is accumulated by Brownian and thermophoretic diffusion. Soret and Dufour's effects are considered. Current density is given by J=σ(E+V×B). Electric field strength is neglected. The heat transmission rate is explored via heat source/sink and thermal radiation. Induced magnetic field and Hall effects are neglected due to the low magnetic Reynolds number. Constant magnetic field $B_{0}$ is applied normally to flow direction. Geometry of flow model is given in Fig. (1).

After implementing boundary layer assumptions and problem-related assumptions, we get (see Refs. [19,33])

(1) $$\frac{\partial u}{\partial r}+\frac{u}{r}+\frac{\partial w}{\partial z}=0\text{,}$$

(2) $$\begin{array}{l} u\frac{\partial u}{\partial r}+w\frac{\partial u}{\partial z}=\nu_{f}\frac{\partial^{2}u}{\partial z^{2}}+\frac{\alpha_{1}}{\rho_{f}}\left\{u\left(\frac{\partial^{3}u}{\partial r\partial z^{2}}\right)+w\left(\frac{\partial^{3}u}{\partial z^{3}}\right)+\left(2\frac{\partial w}{\partial z}+3\frac{\partial u}{\partial r}\right)\left(\frac{\partial^{2}u}{\partial z^{2}}\right)+\left(4\frac{\partial^{2}u}{\partial r\partial z}+\frac{\partial^{2}w}{\partial z^{2}}\right)\left(\frac{\partial u}{\partial z}\right)\right\}\;+\frac{\alpha_{2}}{\rho_{f}}\left\{\left(4\frac{\partial^{2}u}{\partial r\partial z}+\frac{\partial^{2}w}{\partial z^{2}}+\frac{1}{r}\left(\frac{\partial u}{\partial z}\right)\right)\left(\frac{\partial u}{\partial z}\right)+\left(\frac{\partial u}{\partial r}+\frac{\partial w}{\partial z}\right)\left(\frac{2\partial^{2}u}{\partial z^{2}}\right)\right\}+\frac{\beta_{3}}{\rho_{f}}\{\left(\frac{4}{r}{\left(\frac{\partial w}{\partial z}\right)}^{2}+16\frac{\partial u}{\partial z}\frac{\partial^{2}u}{\partial r\partial z}\right)\left(\frac{\partial u}{\partial r}\right)\\ +8\left(\frac{\partial u}{\partial z}\frac{\partial w}{\partial z}\frac{\partial^{2}w}{\partial z^{2}}\right)+\left(4\frac{\partial^{2}u}{\partial r^{2}}+6\frac{u}{r^{2}}+2\frac{\partial^{2}w}{\partial r\partial z}\right){\left(\frac{\partial u}{\partial z}\right)}^{2}+\left({\left(\frac{\partial u}{\partial r}\right)}^{2}+4{\left(\frac{u}{r}\right)}^{2}+4{\left(\frac{\partial w}{\partial z}\right)}^{2}+4\frac{\partial w}{\partial r}\frac{\partial u}{\partial z}\right)\left(\frac{\partial^{2}u}{\partial z^{2}}\right)\}\\ \;-\frac{\sigma^{*}}{\rho_{f}}B_{0}^{2}\;\sin\;\alpha^{*}u\text{,} \end{array}\}$$

(3) $$u\frac{\partial T}{\partial r}+w\frac{\partial T}{\partial z}=\left(\frac{k}{{\left(\rho c_{p}\right)}_{f}}+\frac{16\sigma^{*}T_{\infty }^{3}}{3k^{*}{\left(\rho c_{p}\right)}_{f}}\right)\left(\frac{\partial^{2}T}{\partial z^{2}}\right)+\tau \left(D_{B}\left(\frac{\partial C}{\partial z}\frac{\partial T}{\partial z}\right)+\frac{D_{T}}{T_{\infty }}{\left(\frac{\partial T}{\partial z}\right)}^{2}\right)+\frac{\sigma^{**}B_{0}^{2}}{{\left(\rho c_{p}\right)}_{f}}\sin\;\alpha^{*}u^{2}+\frac{1}{{\left(\rho c_{p}\right)}_{f}}Q_{0}\left(T-T_{\infty }\right)+\frac{D_{B}k_{T}}{c_{s}{\left(c_{p}\right)}_{f}}\left(\frac{\partial^{2}C}{\partial z^{2}}\right)\}$$

(4) $$u\frac{\partial C}{\partial r}+w\frac{\partial C}{\partial z}=D_{B}\left(\frac{\partial^{2}C}{\partial z^{2}}\right)+\left(\frac{D_{T}}{T_{\infty }}+\frac{D_{B}k_{T}}{T_{m}}\right)\left(\frac{\partial^{2}T}{\partial z^{2}}\right)-K_{r}^{2}\left(C-C_{\infty }\right){\left(\frac{T}{T_{\infty }}\right)}^{n}e^{\left(\frac{-E_{a}}{KT}\right)}\text{,}$$

with

(5) $$\begin{array}{l} \mu (T)\frac{\partial u}{\partial z}=\frac{\partial \sigma }{\partial T}\frac{\partial T}{\partial r}+\frac{\partial \sigma }{\partial C}\frac{\partial C}{\partial r},T_{w}=\frac{T_{0}r^{2}}{r^{*2}},C_{w}=\frac{C_{0}r^{2}}{r^{*2}},w=0\;\text{at}\;z=0\text{,}\\ u\to 0,C\to C_{\infty },T\to T_{\infty }\;\text{as}\;z\to \infty \text{.} \end{array}\}$$

Surface tension is defined as [34,33]

(6) $$\sigma =\sigma_{0}-\gamma_{C}\left(C-C_{\infty }\right)-\gamma_{T}\left(T-T_{\infty }\right)\text{,}$$

(7) $$\text{where}\;\gamma_{C}={-\frac{\partial \sigma }{\partial C}|}_{C\to C_{\infty }}\text{and}\;\gamma_{T}={-\frac{\partial \sigma }{\partial T}|}_{T\to T_{\infty }}\text{.}$$

Note that $\gamma_{C},\gamma_{T}$, and $\sigma_{0}$ are positive constants.

Considering the following transformations

(8) $$\begin{array}{l} u=arf_{\eta }\left(\xi ,\eta \right)\;w=-\sqrt{a\nu }\left\{2f\left(\xi ,\eta \right)+\xi f_{\xi }\left(\xi ,\eta \right)\right\}\text{,}\\ \theta \left(\xi ,\eta \right)=\frac{\left(T-T_{\infty }\right)r^{*2}}{T_{0}r^{2}},\phi \left(\xi ,\eta \right)=\frac{\left(C-C_{\infty }\right)r^{*2}}{C_{0}r^{2}},\eta =\sqrt{\frac{a}{\nu }}z,\xi =\frac{r}{r^{*}}\text{,} \end{array}\}$$

Using these transformations in Eqs. (1), (2), (3), (4), (5), (6), (7), (8), and applying first order truncation, we get

(9) $$f_{\eta \eta \eta }-{f_{\eta }}^{2}+ff_{\eta \eta }+M_{1}\left\{{f_{\eta \eta }}_{\eta }\left(1-4f+3f_{\eta }\right)-2f{f_{\eta \eta }}_{\eta \eta }-f_{\eta \eta }^{2}\right\}+M_{2}\left\{3f_{\eta \eta }^{2}-2f_{\eta }f_{\eta \eta \eta }\right\}+M_{3}\left\{58f_{\eta }f_{\eta \eta }^{2}+37f_{\eta }^{2}f_{\eta \eta \eta }+16f_{\eta }^{3}\right\}-M\;\sin\;\alpha^{*}f_{\eta }=0\text{,}\}$$

(10) $$\left(1+Rd\right)\theta_{\eta \eta }-\mathrm{Pr}\left(2\;\mathrm{Pr}\;f_{\eta }\theta -2f\theta_{\eta }\right)+\mathrm{Pr}\;N_{t}\left({\theta_{\eta }}^{2}\right)+\mathrm{Pr}\;N_{b}\left(\theta_{\eta }\phi_{\eta }\right)+\mathrm{Pr}\;N_{t}\left({\theta_{\eta }}^{2}\right)+\mathrm{Pr}\;EcM\;\sin\;\alpha^{*}\left({f_{\eta }}^{2}\right)+\mathrm{Pr}\;\delta \left(\theta \right)+\mathrm{Pr}\;Du\left({\phi_{\eta }}_{\eta }\right)=0\text{,}\}$$

(11) $${\phi_{\eta }}_{\eta }+Sc\left(2f\phi_{\eta }-2f_{\eta }\phi \right)+\left(\frac{N_{t}}{N_{b}}+SrSc\right){\theta_{\eta }}_{\eta }-Sck_{1}\left(\phi \right){\left(1+\Omega \xi^{2}\theta \right)}^{n}e^{\left(\frac{-E_{1}}{\left(1+\Omega \xi^{2}\theta \right)}\right)}=0\text{,}$$

with

(12) $$f\left(0\right)=0,\theta \left(0\right)=1,f_{\eta \eta }\left(0\right)=-2Ma\left(1+Ra\right),\phi \left(0\right)=1,\theta \left(\infty \right)=0,f_{\eta }\left(\infty \right)=0,\phi \left(\infty \right)=0\text{.}$$

Related parameters are

(13) $$\begin{array}{l} M_{1}=\frac{\alpha_{1}a}{\rho \upsilon },M_{2}=\frac{\alpha_{2}a}{\rho \upsilon },M_{3}=\frac{\beta_{3}a^{2}}{\rho \upsilon },M=\frac{\sigma^{*}B_{0}^{2}}{\rho a},\mathrm{Pr}=\frac{\upsilon }{\alpha },Sc=\frac{\nu }{D_{B}},Ec=\frac{a^{2}{r^{*}}^{2}}{c_{p}T_{0}}\text{,}\\ Sr=\frac{D_{B}k_{T}T_{0}}{\upsilon C_{0}T_{m}},Du=\frac{D_{T}k_{T}C_{0}}{\nu T_{0}c_{s}c_{p}},N_{t}=\frac{\tau D_{T}T_{0}}{\upsilon T_{\infty }},N_{b}=\frac{\tau D_{B}C_{0}}{\upsilon },Rd=\frac{16\sigma^{*}}{3kk^{*}}T_{\infty }^{3},\delta =\frac{Q_{0}}{a\left(\rho c_{p}\right)}\text{,}\\ Ma=\frac{\gamma_{T}T_{0}}{\mu_{f}ar^{*2}}\sqrt{\frac{\upsilon }{a}},Ra=\frac{T_{0}\gamma_{C}}{C_{0}\gamma_{T}},k_{1}=\frac{k_{r}^{2}}{a},\Omega =\frac{T_{0}}{T_{\infty }},E_{1}=\frac{E_{a}}{KT_{\infty }}\text{.} \end{array}\}$$

### 2.1 Entropy analysis

Entropy is given as

(14) $$E_{G}=\frac{k}{T_{\infty }^{2}}\left(1+\frac{16\sigma^{*}}{3k^{*}k}T_{\infty }^{3}\right)\;{\left(\frac{\partial T}{\partial z}\right)}^{2}+\frac{R_{D}}{C_{\infty }}{\left(\frac{\partial C}{\partial z}\right)}^{2}+\frac{R_{D}}{T_{\infty }}\left(\frac{\partial T}{\partial z}\frac{\partial C}{\partial z}\right)+\frac{\sigma^{*}}{T_{\infty }}B_{0}^{2}\;\sin\;\alpha^{*}u^{2}\text{,}$$

(15) $$S_{G}=\Omega \left(1+Rd\right){\theta_{\eta }}^{2}+\frac{L\Lambda }{\Omega }{\phi_{\eta }}^{2}+L\theta_{\eta }\phi_{\eta }+MBr\;\sin\;\alpha^{*}{f_{\eta }}^{2}\text{.}$$

where

(16) $$Bejan\;number=\frac{Entropy\;via\;heat\;and\;mass\;transfer}{Total\;entropy}\text{,}$$

(17) $$Be=\frac{\Omega \left(1+Rd\right){\theta_{\eta }}^{2}+L\frac{\Lambda }{\Omega }{\phi_{\eta }}^{2}+L\theta_{\eta }\phi_{\eta }}{\Omega \left(1+Rd\right){\theta_{\eta }}^{2}+MBr\;\sin\;\alpha^{*}{f_{\eta }}^{2}+L\theta_{\eta }\phi_{\eta }+\frac{L\Lambda }{\Omega }{\phi_{\eta }}^{2}.}\text{,}$$

where

(18) $$L=\frac{R_{D}C_{0}}{k},S_{G}=\frac{E_{G}\upsilon T_{\infty }}{k\alpha T_{0}},\Lambda =\frac{C_{0}}{C_{\infty }}\text{.}$$

### 2.2 Physical quantities

#### 2.2.1 Heat transfer rate

Local Nusselt number is

(19) $$Nu_{r}=\frac{rq_{w}}{k\left(T_{w}-T_{\infty }\right)}\text{,}$$

where heat flux $\left(q_{w}\right)$ is

(20) $$q_{w}={-k\left(1+\frac{16\sigma^{*}}{3k^{*}k}T_{\infty }^{3}\right)\left(\frac{\partial T}{\partial z}\right)|}_{z=0}\text{,}$$

In dimensionless form

(21) $${Re}_{r}^{-1/2}Nu_{r}=-\left(1+Rd\right)\theta_{\eta }\left(0\right)\text{.}$$

#### 2.2.2 Mass transfer rate

Sherwood number is expressed as

(22) $$Sh_{r}=\frac{rJ_{w}}{D_{B}\left(C_{w}-C_{\infty }\right)}\text{,}$$

where the mass flux $\left(J_{w}\right)$ is

(23) $$J_{w}={-D_{B}\left(\frac{\partial C}{\partial z}\right)|}_{z=0}\text{,}$$

In dimensionless form

(24) $$Re_{r}^{-1/2}Sh_{r}=-\phi_{\eta }\left(0\right)\text{.}$$

## 3 Solution methodology

The PDEs associated to the problem are non-dimensionalized via adequate variables. The non-dimensional PDEs are then tackled through ND-solve. To ensure convergence of ND-solve, an appropriate numerical method with fine grid, initial and boundary conditions are required. Note that in above equations, *f*, *θ, ϕ, S_G_* and *Be* are functions of *ξ* and *η.For* solutions purpose we have taken *ξ=1* and treated *f, θ, ϕ, S_G_ and Be* as funtions of *η* (see all plots)*.*

## 4 Analysis

Effects of the Marangoni number, Marangoni ratio parameter, magnetic, radiation, heat generation, diffusion, and other involved influential flow parameters on the velocity field, temperature distribution, concentration, Bejan number and entropy rate are presented below. Table 1 is created to provide naming for the various parameters and expressions involved in our study.

**Table 1:** Nomenclature for the problem under consideration.

Br	Brinkman number	$\nu_{f}$	fluid kinematic viscosity
T	Temperature	u,w	components of velocity along r,z direction
$N_{t}$	thermophoresis parameter	Ra	Marangoni ratio parameter
$K_{r}$	reaction rate	$c_{s}$	concentration succesibility
Sc	Schmidt number	$C_{\infty }$	ambient concentration
$E_{a}$	activation energy coefficient	$T_{0}$	reference temperature
$\alpha^{*}$	inclined angle	$T_{w}$	surface temperature
Ma	Marangoni number	$\mu_{f}$	fluid dynamic viscosity
δ	heat generation parameter	Ω	temperature difference parameter
$C_{w}$	surface concentration	f	dimensionless velocity profile
k	thermal conductivity	θ	dimensionless temperature profile
$\rho_{f}$	density of fluid	C	Concentration
L	diffusion parameter	$E_{1}$	activation energy parameter
$B_{o}$	magnetic field strength	$Nu_{x}$	Nusselt number
M	magnetic parameter	$k_{1}$	chemical reaction parameter
ϕ	dimensionless concentration profile	$S_{G}$	entropy rate
$c_{p}$	specific heat	Λ	concentration ratio parameter
Sr	Soret number	$\sigma^{**}$	Stephan-Boltzmann constant
$Sh_{x}$	Sherwood Number	Pr	Prandtl number
$Q_{o}$	heat source coefficient	Du	Dufour number
$k^{*}$	mean absorption coefficient	A,B	positive constants
Ec	Eckert number	$T_{\infty }$	ambient temperature
$D_{B}$	Brownian motion coefficient	$D_{T}$	thermophoretic diffusion coefficient
Rd	radiation parameter	${Re}_{r}$	local Reynold number
$\left(a_{1},a_{2},\beta_{3}\right)$	fluid material coefficients	τ	ratio of heat capacities
$\left(M_{1},M_{2},M_{3}\right)$	third grade fluid parameters	${\left(\rho c_{p}\right)}_{nf}$	nanofluid heat capacity
${\left(\rho c_{p}\right)}_{f}$	fluid heat capacity	$N_{b}$	Brownian motion parameter
*kT*	Ratio of thermal diffusions	σ*	Electrical conductivity
*μ(T)*	Varible viscosity	*K*	Chemical reaction coefficient

### 4.1 Velocity

**Fig. (2):**
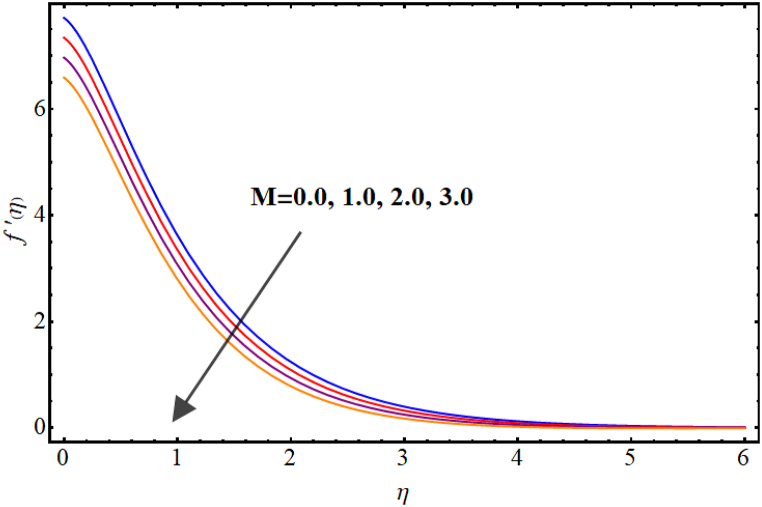
M vs Velocity.

**Fig. (3):**
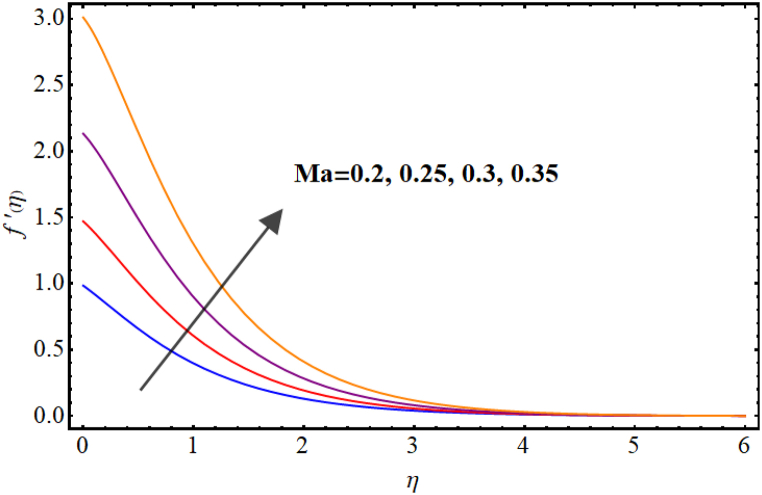
Ma vs Velocity.

**Fig. (4):**
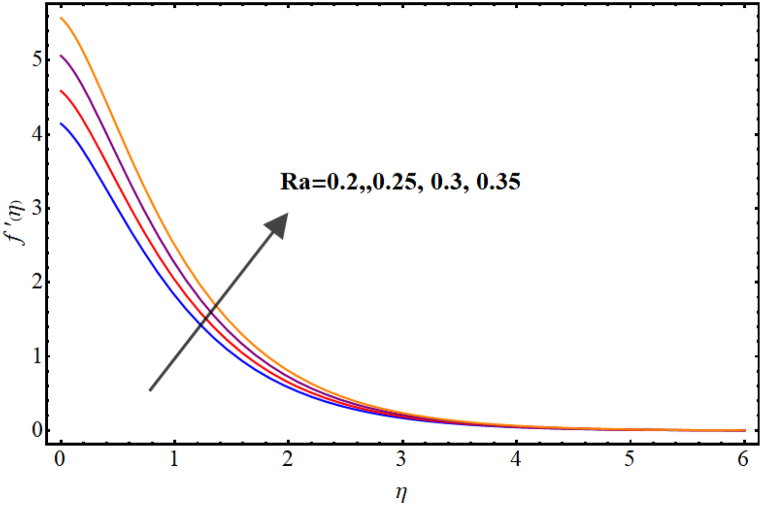
Ra vs Velocity.

**Fig. (5):**
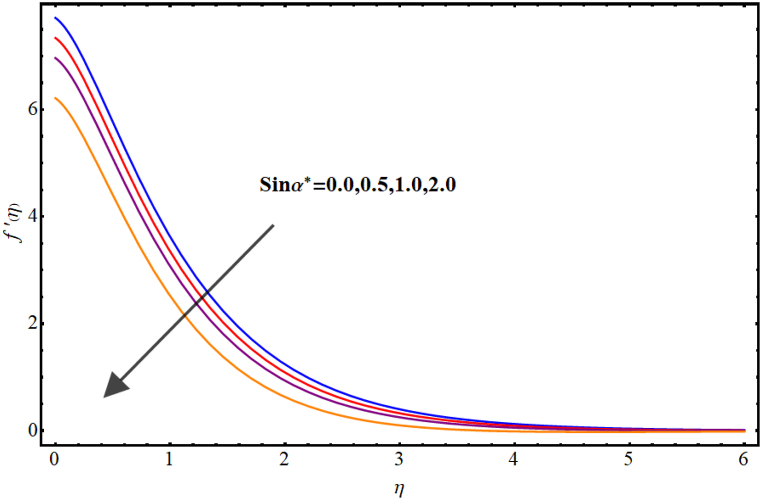
sin $\propto^{*}$vs Velocity.

Velocity variations over various flow variables are presented in Fig. (2), Fig. (3), Fig. (4), Fig. (5). Here, the influence of the magnetic parameter on the velocity field is illustrated in Fig. (2). Velocity reduces against higher magnetic parameter. It is due to the enhancement of Lorentz forces in fluid flow which acts as a resistance force to fluid flow and thus the velocity of the fluid decays. Fig. (3) depicts outcomes of Marangoni number (Ma) for fluid velocity. The Marangoni parameter describes the effect of surface tension gradients on fluid flow, and it can have a significant impact on both the velocity and temperature of the fluid. It is clear that the velocity field is enhanced as the value of Marangoni number (Ma) increases. Basically, for higher Marangoni number the ratio of thermal and solutal surface tension increases which enhances fluid flow. An increase in the Marangoni parameter leads to an increase in the velocity of the fluid near the surface, as the surface tension gradients induce a flow that tends to smooth out those gradients. Fig. (4) display influence of Marangoni ratio parameter (Ra) on velocity. An increment in Marangoni ratio parameter (Ra) results in velocity enhancement. Fig. (5) Witnesses velocity variations for $\sin\;\alpha^{*}$. Here, velocity decays against higher $\sin\;\alpha^{*}$.

### 4.2 Temperature

**Fig. (6):**
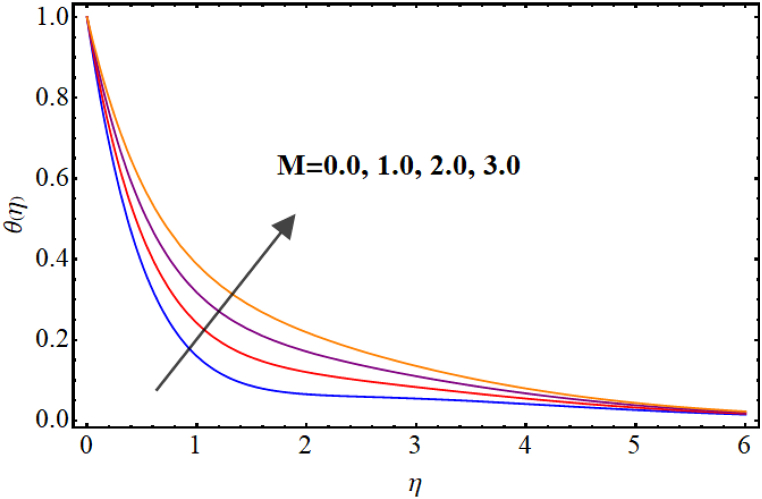
M vs Temperature.

**Fig. (7):**
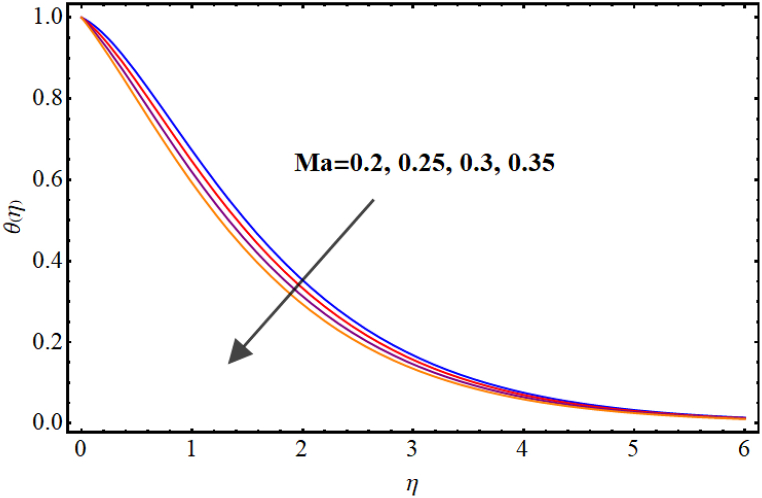
Ma vs Temperature.

**Fig. (8):**
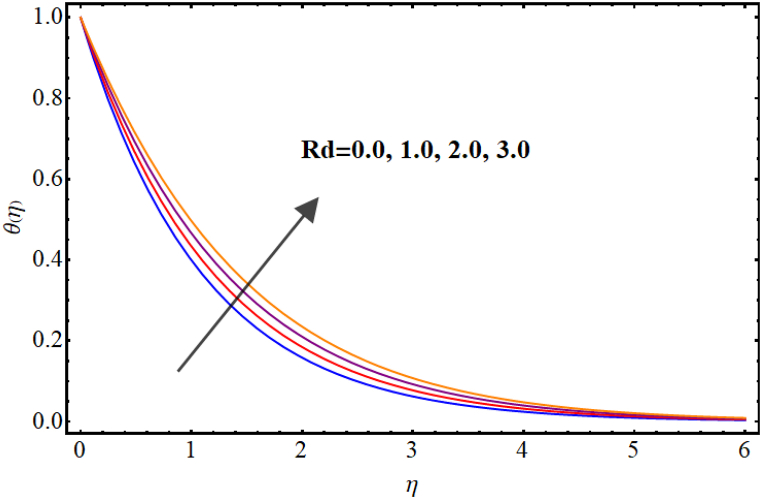
Rd vs Temperature.

**Fig. (9):**
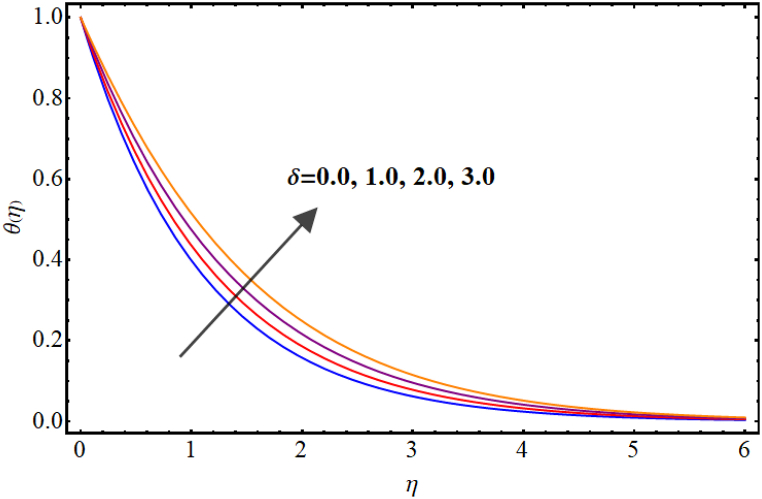
δ**vs Temperature.**

**Fig. (10):**
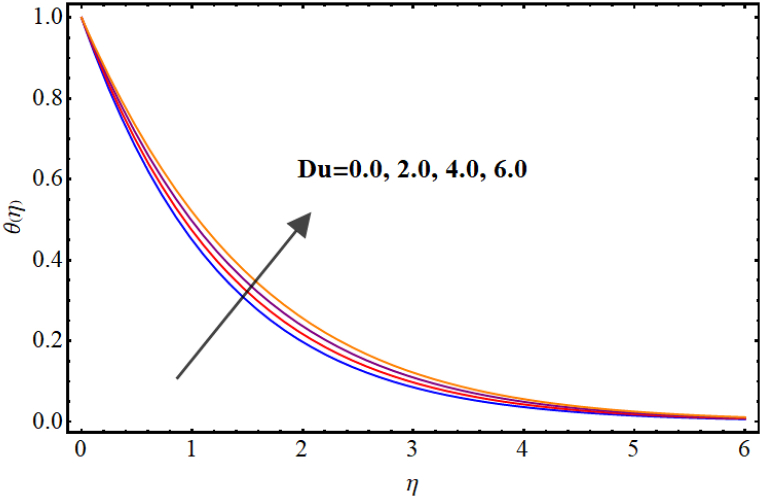
Du vs Temperature.

**Fig. (11):**
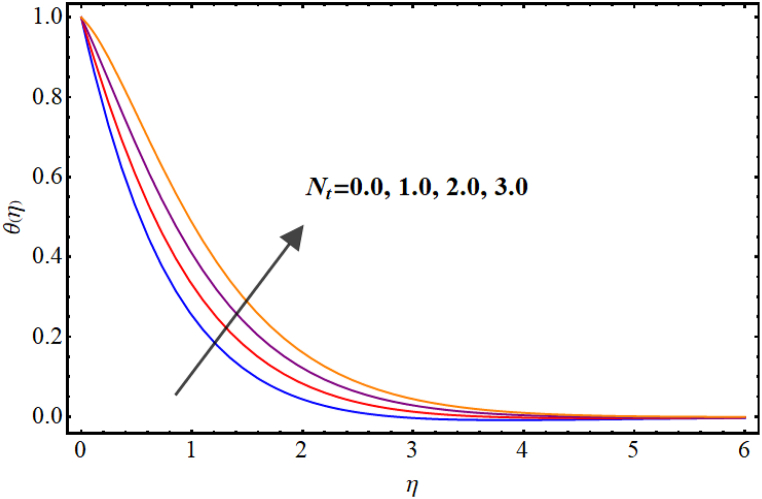
$N_{t}$**vs Temperature.**

Temperature variations for different involved parameters are depicted in Fig. (6), Fig. (7), Fig. (8), Fig. (9), Fig. (10), Fig. (11). Fig. (6) displays impact of magnetic parameter on temperature. Temperature (θ(η)) rises for higher magnetic parameter (M). Physically, for larger (M) due to strong resistive forces (Lorentz force) cause resistance to liquid flow and results in more collision between the fluid particles thus temperature boosts for higher (M). Temperature variations for Marangoni number (Ma) is displayed in Fig. (7). A reduction in temperature (θ(η)) occurs for higher estimations of Marangoni number (Ma). Impact of radiation on temperature is displayed in Fig. (8). Higher (Rd) results in temperature enhancement. Physically, for an augmentation in radiation causes a decline in mean absorption coefficient which enhances thermal flux and thus temperature improves. Influence of heat generation (δ) on temperature distribution is illustrated in Fig. (9). (θ(η)) increases against higher heat generation parameter (δ). When a heat source is present, it releases thermal energy into the system, which raises the internal energy of the fluid and leads to an increase in temperature. The amount of temperature increase will depend on the amount of heat released, the heat capacity of the fluid, and the size of system. A rise in temperature can be seen for higher values of Dufour number (Du) see Fig. (10). Effects of thermophoresis parameter ($N_{t}$) on (θ(η)) is given in Fig. (11). A rise in temperature occurs for large values of thermophoresis parameter ($N_{t}$).

### 4.3 Concentration

**Fig. (12):**
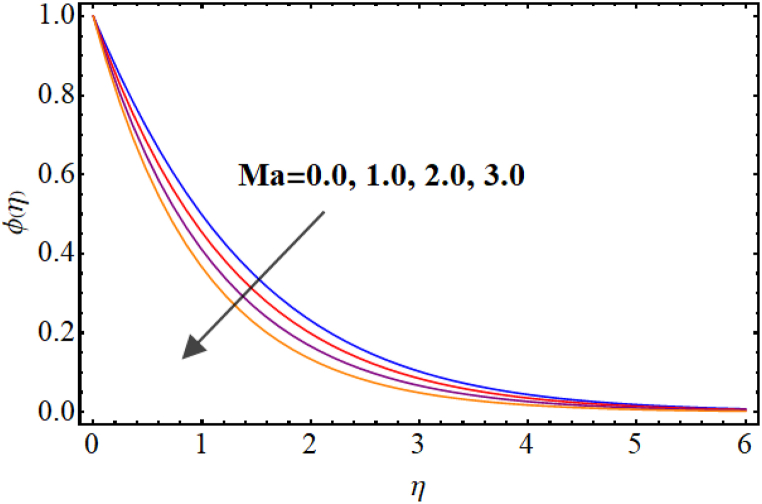
Ma vs Concentration.

**Fig. (13):**
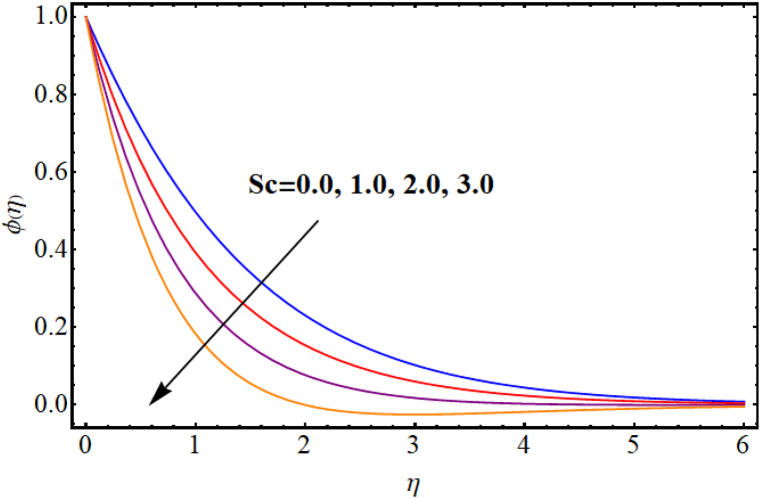
Sc vs Concentration.

**Fig. (14):**
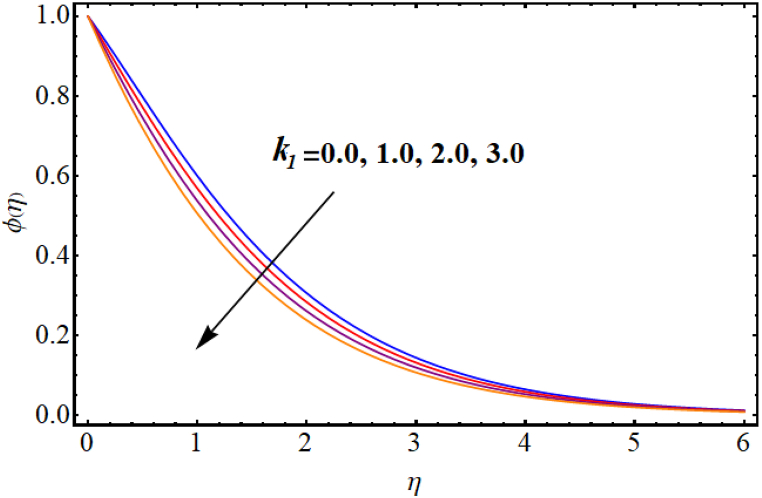
$k_{1}$**vs Concentration.**

**Fig. (15):**
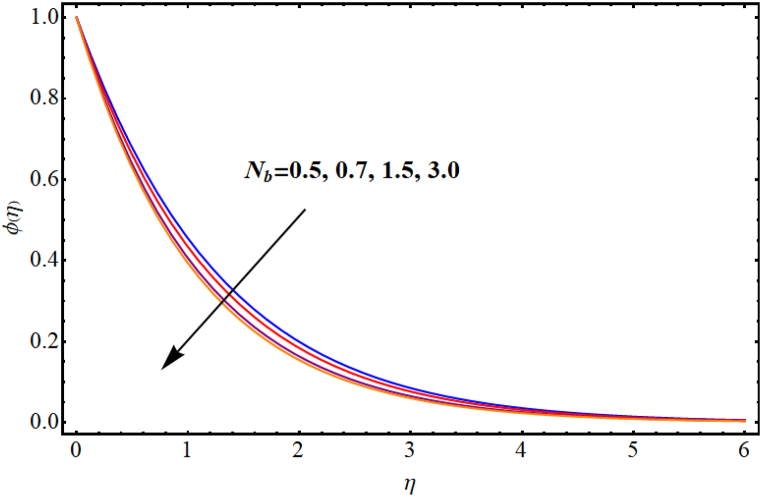
$N_{b}$**vs Concentration.**

**Fig. (16):**
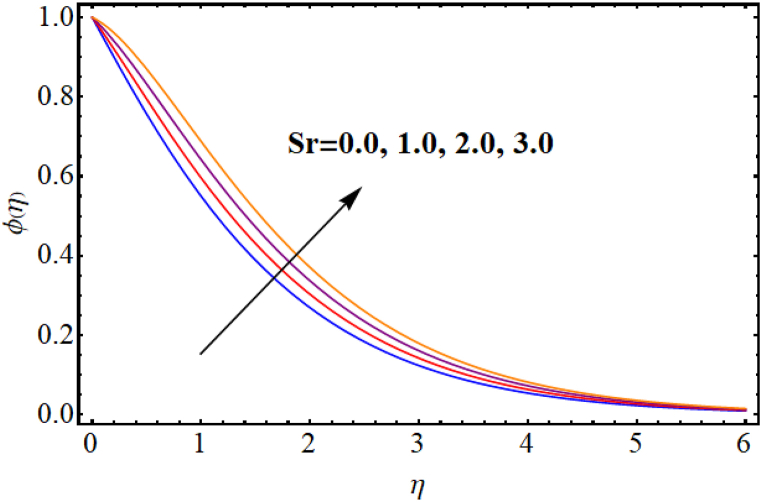
Sr vs Concentration.

**Fig. (17):**
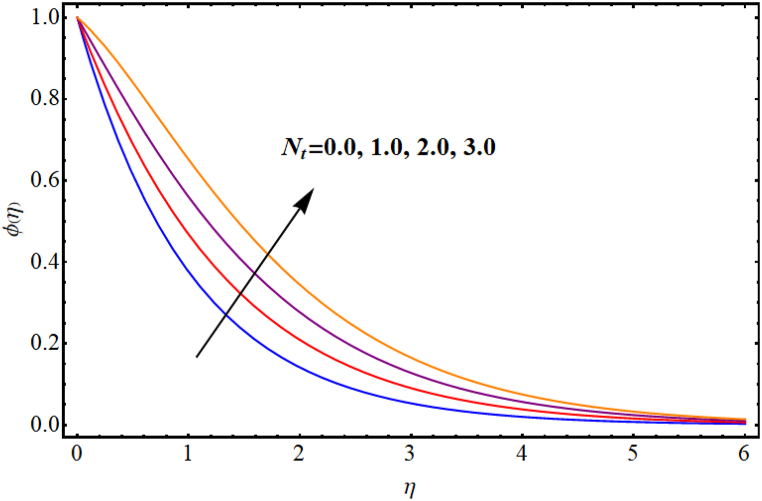
$N_{t}$**vs Concentration.**

Fig. (12), Fig. (13), Fig. (14), Fig. (15), Fig. (16), Fig. (17) expresses the impacts of different flow parameters of concentration. Fig. (12) represents the effect of Marangoni number (Ma) on concentration. Clearly, concentration decays against higher Marangoni number (Ma). Fig. (13) illustrate variation of concentration for Schmidt number (Sc). Concentration reduces for higher Schmidt number. Since, mass diffusivity decays for an increment in Schmidt number and as a result reduces. Fig. (14) depicts effect of ($k_{1}$) over concentration. ($\phi \left(\eta \right)$) decreases against higher chemical reaction parameter ($k_{1}$). Fig. (15) witnesses’ outcome of Brownian motion parameter ($N_{b}$) for concentration. Reduction in concentration occurs against higher Brownian motion parameter ($N_{b}$). Brownian diffusion is the process by which particles in a fluid move randomly due to collisions with other particles. This can lead to a decrease in concentration of the particles over time, as the particles become dispersed throughout the fluid. Fig. (16) elucidate influence of Soret number (Sr) for concentration. Concentration enlargement occurs for higher Soret number (Sr). Fig. (17) display variation of concentration against thermophoresis parameter ($N_{t}$). An increment in concentration is observed for higher thermophoresis parameter ($N_{t}$).

### 4.4 Bejan number and entropy generation

**Fig. (18):**
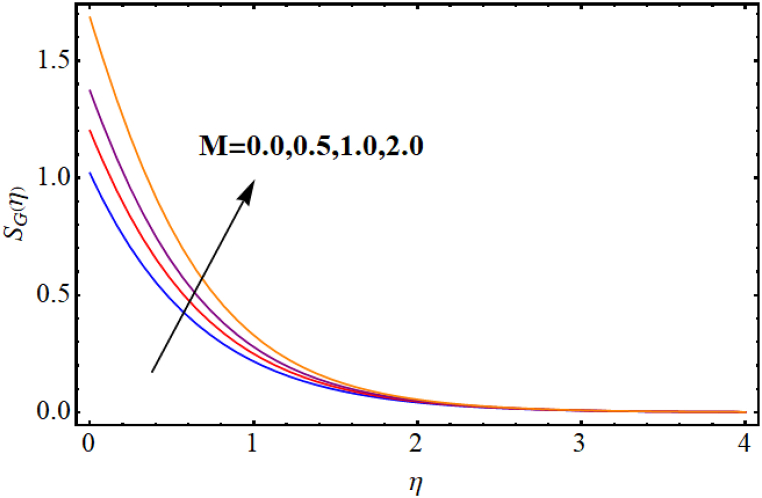
M vs Entropy rate.

**Fig. (19):**
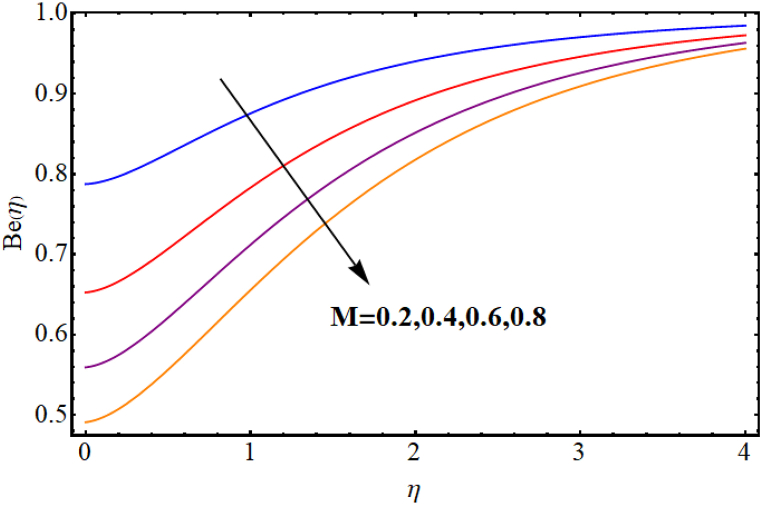
M vs Bejan number.

**Fig. (20):**
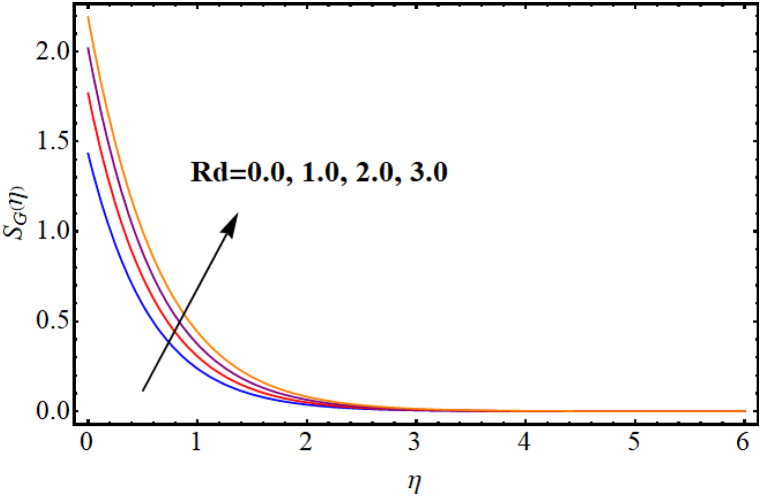
Rd vs Entropy rate.

**Fig. (21):**
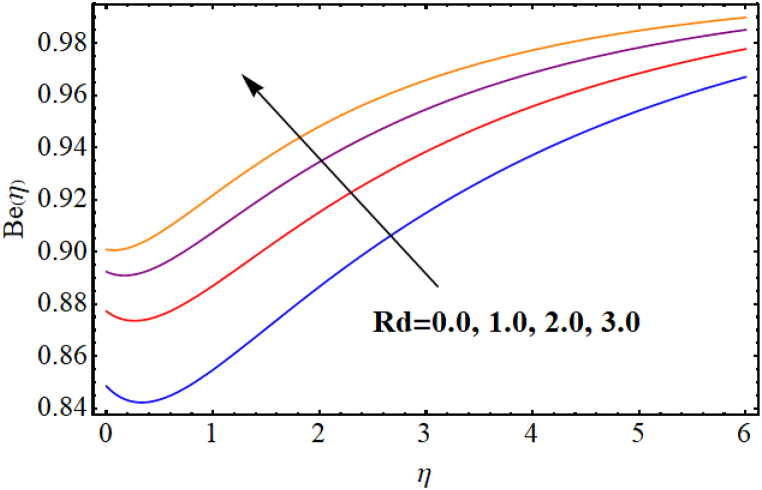
Rd vs Bejan number.

**Fig. (22):**
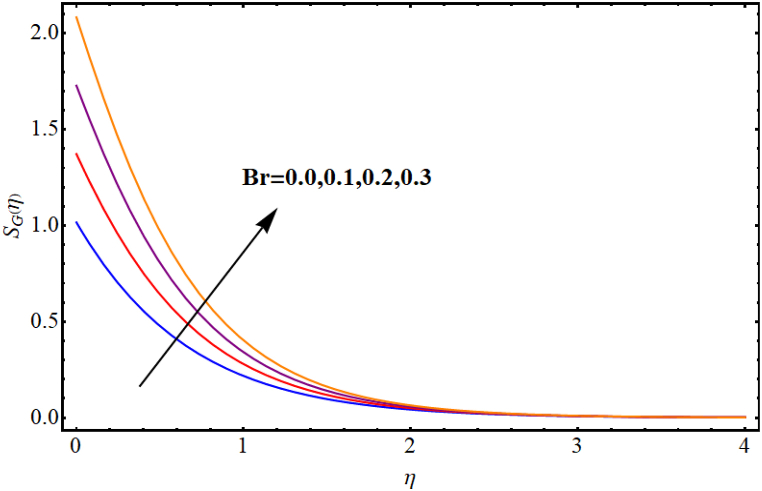
Br vs Entropy rate.

**Fig. (23):**
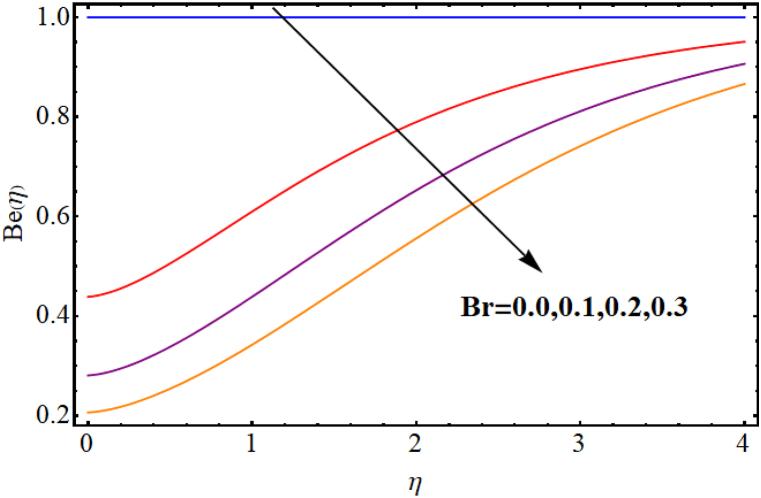
Br vs Bejan number.

**Fig. (24):**
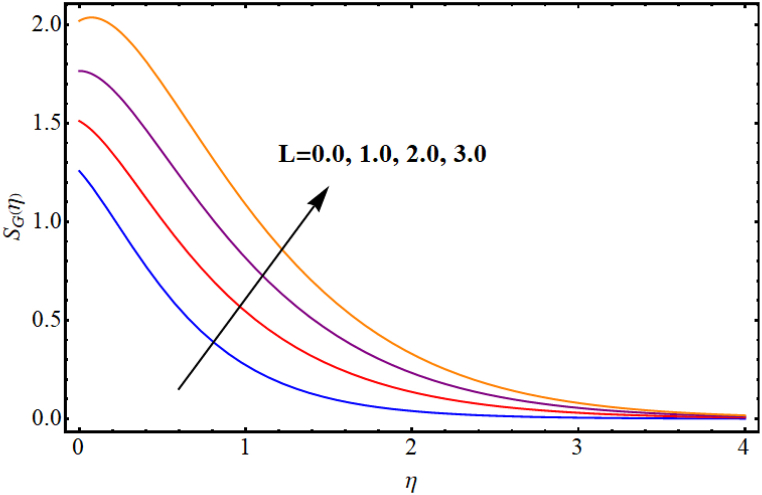
L vs Entropy rate.

**Fig. (25):**
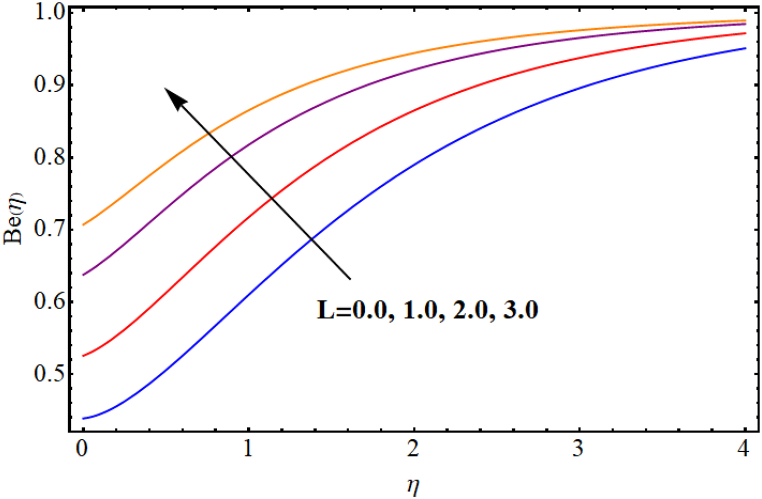
L vs Bejan number.

The entropy generation rate and Bejan number outcomes for various parameters are displayed in Fig. (18), Fig. (19), Fig. (20), Fig. (21), Fig. (22), Fig. (23), Fig. (24), Fig. (25). Fig. (18) depicts impact of magnetic parameter on entropy rate ($S_{G}\left(\eta \right)$). An increment in magnetic parameter (M) results in entropy rate enhancement. Physically, more resistive forces (Lorentz forces) generate for higher magnetic field. Which enhances system disorder and consequently entropy rate boosts. Variations of magnetic field for Bejan number (Be) is displayed in Fig. (19). Reduction in Bejan number occurs for larger magnetic parameter (M). Fig. (20), Fig. (21) are intended to display impact of radiation on ($S_{G}\left(\eta \right)$) and (Be). Both entropy rate and Bejan number show similar behavior against higher radiation parameter (Rd). Physically an increment in (Rd) results in more radiation emission which increases disorder in system and as a result entropy rate increase. Fig. (22), Fig. (23) display effect of Brinkman number (Br) on entropy rate ($S_{G}\left(\eta \right)$) and Bejan number (Be). Reverse behavior of entropy rate and Bejan number is noted against higher Brinkman number. Entropy rate boosts while a decline in Bejan number occurs against higher Brinkman number (Br). Basically, Brinkman is concerned with dissipation effects and an increment in Brinkman number results in dominant viscous effect and as a result system entropy increase. Fig. (24), Fig. (25) elucidate outcomes of diffusion parameter (L) for entropy rate and Bejan number. Clearly, both entropy rate and Bejan number boosts against higher diffusion parameter (L).

## 5 Final remarks

In the present work, our main intention was to examine the irreversibility analysis with the transportation of Marangoni convection in third-grade nanofluid with Dufour and Soret effects. Joule heating, thermal radiation, and internal heat generation described heat transport features, while the chemical reaction and activation energy was considered to determine mass transport characteristics. The PDEs governed are non-dimensionalized via suitable variables. Effects of different flow parameters on quantities of interest were explored graphically. The main observations regarding the presented work are listed below.

➢ The velocity field increased due to increment in the Marangoni number (Ma).
➢ The velocity field was reduced via magnetic number (M) and $\sin\;\alpha^{*}$.
➢ The temperature field rises with an increase in Dufour number (Du) and radiation parameter (Rd).
➢ Decrease in the thermal transport occurs for large value of Marangoni number (Ma).
➢ The concentration profile enhances for large values of Soret number (Sr).
➢ Reduction in concentration was observable against chemical reaction and Brownian motion parameters.
➢ Irreversibility ratio distribution and entropy rate show opposite behavior against Brinkman number (Br).
➢ Entropy generation was augmented due to increment in the diffusion parameter (L).
➢ Bejan number boosted for large value of the diffusion parameter (L).

## Author contribution statement

K. Muhammad: Contributed analysis tools or data; Wrote the paper.

T. Hayat: Analyzed and interpreted the data; Wrote the paper.

Inayatullah, S. Momani: Conceived and designed the analysis; Wrote the paper.

## Data availability statement

Data will be made available on request.

## Declaration of competing interest

The authors declare that they have no known competing financial interests or personal relationships that could have appeared to influence the work reported in this paper.
